# Expression signatures and roles of MicroRNAs in human oesophageal adenocarcinomas

**DOI:** 10.1111/jcmm.13300

**Published:** 2017-08-10

**Authors:** Xiangyi Kong, Shun Gong, Lijuan Su, Chen Li, Yanguo Kong

**Affiliations:** ^1^ Department of Neurosurgery Peking Union Medical College Hospital Chinese Academy of Medical Sciences Beijing China; ^2^ Department of Breast Oncology National Cancer Center/Cancer Hospital Peking Union Medical College Chinese Academy of Medical Sciences Beijing China; ^3^ Department of Neurosurgery Shanghai Institute of Neurosurgery PLA Institute of Neurosurgery Shanghai Changzheng Hospital Second Military Medical University Shanghai China; ^4^ College of Computer Science and Technology Zhejiang University Hangzhou Zhejiang China; ^5^ Cancer Epigenetic Laboratory Department of Clinical Oncology State Key Lab of Oncology in South China Sir YK Pao Center for Cancer Li Ka Shing Institute of Health Science The Chinese University of Hong Kong Shatin Hong Kong

**Keywords:** oesophageal adenocarcinomas, Barrett's oesophagus, expression, microRNA

## Abstract

The most common forms of oesophageal cancers are adenocarcinomas and squamous cell carcinoma (SCC). Although the incidence of SCC in the United States tends to be declining, the adenocarcinoma incidence caused by Barrett's oesophagus has been increasing. Oesophageal cancer is regarded as one of the most fatal malignancies with a short prognosis. Systemic manifestations of patients with PCNSL keep backward in spite of recent development of chemoradiotherapy. MicroRNAs are small non‐coding RNAs that can post‐transcriptionally down‐regulate the expression of genes by targeting mRNAs, causing their translational repression as well as degradation. MicroRNAs exert critical functions in many malignancy‐related biological processes, including cell apoptosis, metabolism, proliferation and differentiation. Many deregulated miRNAs have been identified in oesophageal adenocarcinomas, but their biological importance has not yet been fully elucidated. In this study, we review present evidence regarding the potential applications of oesophageal adenocarcinomas associated microRNAs for prognosis and diagnosis of this lethal disease.

## Introduction

Approximately 16,910 cases of oesophageal cancer are diagnosed each year in the United States, with approximately 15,690 deaths from the disease 1. In 2012, there were approximately 455,800 new cases of oesophageal cancer and 400,200 deaths worldwide 2. The incidence rate of oesophageal cancer change globally by almost 16‐fold, with the lowest rates in Western and Middle Africa and Central America, and the highest rates in Eastern and Southern Africa and Eastern Asia in both females and males 3. Squamous cell carcinoma (SCC) comprises a major portion of oesophageal cancers. In the last three decades, the incidence of esophago‐gastric junction (EGJ), oesophageal adenocarcinomas and gastric cardia has been significantly increasing, in both Eastern countries and more frequently in some Western countries as well 4, 5. Several epidemiologic series have demonstrated that the occurrence rate of EAC has exceeded that of the formerly more common SCC. For instance, research into a registry of cancer in the United States predicted that the age‐regulated occurrence rates of EAC increased to 2.5 cases per 100,000 people during 1992–1996, from 1.8 cases per 100,000 people in 1987–1991 6. Men were diagnosed eight times more often than women, and white males were affected five times more frequently than black males, although the occurrence rate among white women has also risen 6.

A remarkable growth in the incidence of EAC was observed among persons aged of 45–65 years. Several studies have described risk factors associated with EAC. Approximately 80% of EAC cases were associated with smoking history, gastroesophageal reflux disease, body mass index higher than the lowest quartile, and a diet that was low in vegetables and fruits 7, 8. Much more is acquainted on the early pathology of EAC due to identification of early cancer when surveillance of patients with Barrett's oesophagus (BE) 9. Although the molecular biological mechanisms of BE and its evolution to EAC have been broadly researched, the molecular mechanisms underlying BE‐EAC carcinogenesis have not yet been entirely elucidated. The identification of effective biomarkers for early diagnosis of EAC and a better understanding of the systems of the neoplastic advancement are consequently keenly awaited.

## MicroRNA and cancers

MicroRNAs (miRNAs) are small (18–25 nucleotides) RNAs with non‐protein‐coding capacity that function in regulating the expression of genes 10. Maturation and synthesis of miRNAs involve a stepwise process in the cytoplasm and the nucleus 11. In the nucleus, RNA polymerase II transcribes the miRNA transcript, leading to a transcript with a stem‐loop structure of about 50 7‐methylguanylate cap, 70 nucleotides and a 30‐polyadenylated tail 11, 12. miRNAs exert their regulatory roles at the level of post‐transcription by stimulating target messenger RNA (mRNA) degradation and translational repression. Dysregulation of miRNAs has been detected in a great variety of disorders, particularly in malignancies 12. Increased evidence has shown that certain miRNAs function as tumour suppressors or oncogenes in different human cancers 13. In healthy cells, tumour‐suppressive miRNAs are generally expressed and the expressions of oncogenic proteins are down‐regulated, whereas in cancer cells, tumour‐suppressive miRNAs are silenced, resulting in oncogenic protein up‐regulation. Conversely, oncogenic miRNAs are up‐regulated in cancer cells, resulting in the down‐regulation of the expressions of tumour‐suppressive proteins 13, 14. Together these studies demonstrate that miRNAs play significant roles in many pathological and biological processes, including apoptosis, cell proliferations and tumourigenesis.

## miRNAs involved in progression from BE to EAC

Several studies have explored the changes of miRNA expression levels in BE and EAC. Feber *et al*. found that leveraging specific miRNAs could effectively assist with identifying patients who have a relatively high risk of EAC development. More studies in this area have since been published. Smith *et al*. performed q‐PCR analyses and miRNA microarrays for miRNA expressions in EAC, BE with intestinal‐metaplasia, normal gastric epithelia and squamous oesophageal epithelia. The authors identified several differentially expressed miRNAs in BE development and progression to EAC, including miR‐215, miR‐205, miR‐203, miR‐194, miR‐145, miR‐143 and miR‐21. miRNA expression profiles in EAC are shown in Table 1.

**Table 1:** miRNA expression profiles in oesophageal adenocarcinomas (EAC)

No.	Author	Year	Method	Sample	Up‐regulated	Down‐regulated
1	Mathé *et al*.	2009	Quantitative RT‐PCR	Primary EAC tissues	miR‐21, miR‐192, miR‐194, miR‐223‐5p	miR‐203
2	Saad *et al*.	2013	Microarray RT‐PCR	Primary EAC tissues	miR‐7, miR‐21, miR‐192, miR‐194, miR‐196a, miR‐200a, miR‐200b, miR‐215, miR‐429, miR‐574‐5p	miR‐31, miR‐133b, miR‐143, miR‐144, miR‐145, miR‐203, miR‐205, miR‐365, miR‐1274a, miR‐1274b
3	Wu *et al*.	2013	Taqman real‐time PCR‐based profiling	Primary EAC tissues	miR‐17‐3p, miR‐18, miR‐25‐3p, miR‐92, miR‐106	–
4	Revilla‐Nuin *et al*.	2013	Quantitative RT‐PCR	Primary EAC tissues	miR‐192, miR‐194, miR‐196a, miR‐196b	–
5	Warnecke‐Eberz *et al*.	2015	Real‐time PCR, quantitative RT‐PCR	EAC serum	miR‐223‐5p, miR‐483‐5p	miR‐22‐3p, miR‐23b‐5p, miR‐27b‐3p, miR‐149‐5p, miR‐203, miR‐224‐5p, miR‐452‐5p, miR‐671‐3p, miR‐944‐5p, miR‐1201‐5p
6	Slaby *et al*.	2015	Quantitative RT‐PCR	Primary EAC tissues	miR‐192, miR‐194	miR‐23b‐5p, miR‐27b‐3p, miR‐99a, miR‐149‐5p, miR‐203, miR‐205, miR‐210, miR‐378, miR‐4462
7	Drahos *et al*.	2015	Microarray RT‐PCR	Primary EAC tissues	miR‐421, miR‐494, miR‐502‐6p, miR‐575, miR‐630, miR‐663b, miR‐miR‐42286, miR‐miR‐4488, miR‐miR‐4508	–
8	LÜ *et al*.	2016	Microarray RT‐PCR	Primary EAC tissues	Methylation of microRNA‐193b	miR‐193b
9	Zhang *et al*.	2016	PicoGreen and quantitative PCR assays	EAC serum	miR‐25‐3p, miR‐151a‐3p	miR‐100‐5p, miR‐375

### MiR‐21

MiR‐21 is generally up‐regulated in nearly all epithelial‐based solid tumours and plays a key role in many pathways related to apoptosis, thus serving as a well‐established oncogenic miRNA 15. MiR‐21 targets anti‐oncogenes and metastasis‐suppressing genes, like programmed cell death 4 (PDCD4), tumour‐suppressor gene tropomyosin 1 (TPM1), phosphatase and tensin homologue (PTEN) and Sprouty, thereby demonstrating its involvement in cancer growth, invasiveness and metastasis 16. Importantly, compared with squamous oesophageal epithelia, miR‐21 expression is up‐regulated in BE and EAC. Overexpression of miR‐21 in cancerous EAC compared with adjacent non‐cancerous tissues has been established (HR = 4.23; 95% CI = 1.68–10.61), suggesting it may be involved in tumourigenesis in a variety of histological types of oesophageal tumours 17. Whether miR‐21 is actively secreted from cancer cells through exosome pathways or released into circulation continuously through cell apoptosis is still unclear.

### MiR‐196a and miR‐196b

Increased expressions of miR‐196a together with elevated dysplasia in EAC were reported by Maru *et al*. Putative targets of miR‐196a include HMGA family members and HOX genes (HOXD8, HOX C8, HOXA7 and HOXB8) 18. Luthra *et al*. found that miR‐196a, targeting annexin A1 (ANXA1), was up‐regulated in EAC tissue 19. MiR‐196a represses ANXA1 expression, subsequently exerting anti‐apoptotic effects and contributing to EAC cell survival. Luthra *et al*. further found that miR‐196a is a potential biomarker for neoplastic progressions in BE and demonstrated the stepwise up‐regulation of miR‐196a expression during the BE‐EAC transformation 19. Maru *et al*. 20 also found highly increased expressions of miR‐196a in EAC and showed its anti‐apoptotic and growth‐promoting functions. The authors assessed the ability of miR‐196a to function as a biomarker of BE progression to low‐grade dysplasia, high‐grade dysplasia and EAC. Higher expressions of miR‐196a were observed in EAC and BE than in normal squamous mucosa 20. The authors furthermore confirmed that miR‐196a specifically targets S100A9 3’ UTRs, SPRR2C and KRT5 by miR‐196a‐mimic and luciferase reporter‐based assays 20. As BE is the principle identifiable precursor to EAC, miR‐196a level might thus act as a valuable tool in early cancer detection.

The findings by Revilla‐Nuin *et al*. 18 also imply an imperative role for miR‐196b in BE‐connected carcinogenesis. MiR‐196b and miR‐196a are recorded from differentiated genes and differ by one nucleotide. Up‐regulation of miR‐196b has been identified in gastric cancer and leucemia, although in ectopic endometrial tissues its expression is down‐regulated compared with paired eutopic tissues 21, 22. Confirmation of the putative targets of miR‐196b will be critical to learn the detailed biological functions of this miRNA and identify its role in the development of EAC.

### MiR‐192

Previous studies have defined miRNAs related to the advancement of BE to EAC. MiR‐192 is influenced by the p53 tumour suppressor and can induce cell cycle arrest, indicating it functions like a tumour suppressor 23. Revilla‐Nuin *et al*. used quantitative reverse transcription polymerase chain reaction (qRT‐PCR) to perform validation in BE patients who either advanced or did not advance to adenocarcinoma after a minimum 5 years of follow‐up 18. A higher expression of miR‐192 was detected in BE patients who progressed to EAC in contrast to those who did not advance to EAC 18. ROC results showed that in BE miR‐192 level were applicable molecular biomarkers for different patients with BE who are at high danger of processing to EAC 18. Saad *et al*. 24 also found that miR‐192 was up‐regulated in EAC in contrast to BE, up‐regulated in BE close to Barrett's high‐grade dysplasia lesions related to insulted samples of BE, and much higher in EAC phase I than in developed phases (*p*_anova_ ≤ 0.0009, *P*_II&III_ ≤ 0.003 and *P*_I&III_ ≤ 0.006), indicating that miR‐192 may be involved in EAC tumour advancement rather than progression. Nevertheless, in the research by Mathé *et al*. 17, mir‐192 did not show differences in expressions among patients with high stage (TNM = II, III, IV) and low stage (TNM = 0, I) EAC. Hence, more specific different expression traits of miR‐192 in EAC are still required to be surveyed.

## miRNAs associated with diagnosis of EAC

To date, only a few studies have reported the significance of miRNAs derived from EAC tissue as diagnostic biomarkers and evaluated the diagnostic value of miRNAs circulating in blood and other biofluids from EAC patients. Therefore, as the diagnostic potential of miRNAs is seriously underestimated, a substantial study should be performed to develop miRNA‐based markers useful for early diagnosis of EAC. Bansal *et al*. 25 identified five miRNAs with expression levels capable of discriminating between BE patients with dysplasia and BE patients without dysplasia. Feber *et al*. 26 developed a unique miRNA signature associated with high risks of progression from low‐grade to high‐grade EAC and demonstrated utility of a three miRNA‐panel for a highly discriminative prediction of lymph nodal metastases in EAC tumours (Table 1).

### MiR‐203

Hezova *et al*. suggested that miR‐203 may function as a tumour suppressor in EAC because its expression was largely reduced in tumour tissue from EAC patients. Furthermore, miR‐203 also showed a consistent decline in expression in the malignant development from healthy tissues to EAC via BE 15. Saad *et al*. 24 also found an adverse relation between EAC progression and miR‐203 expression levels. Down‐regulation of miR‐203 was largely linked with tumour stage as well as progression in EAC (*P*_I&III_ = 0.01, *P*_II&III_ = 0.002, *P*_I&II_ = 0.054 and *p*_anova_ = 0.0006), suggesting that miR‐203 can target critical pathways linked with cancer progression in EAC 24. Although miR‐203 targets in EAC have not been identified, their down‐regulations were shown in metastatic breast cancer cell lines and have been connected to increased invasion and motility by targeting SNAI2, a transcription factor that improves migration and invasion 27. In skin epithelia, miR‐203 represses and targets the transcription factor p63 28. P63 exerts a critical role in keeping stem cells in layered squamous epithelium 28, 29. MiR‐203‐directed repression of p63 serves to inhibit cellular proliferation capacity and induce cell cycle exit. This suggests that induction of miR‐203 expression is a critical event needed for ending differentiation of squamous cells 30. Whether miR‐203 expression is related to replacement of the oesophageal epithelium is not clear, although miR‐203 expressions are missed in BE, and hence, it is probable that the miR‐203‐directed system of epithelial replacement is missed in these tissues.

### MiR‐26a

MiR‐26a is currently considered as a tumour‐suppressor gene 31. Some studies implied that cell cycle arrest could be induced by ectopic miR‐26a expression in tumour cells at G1 phase through repressing the expression of EZH2 at the post‐transcriptional level 32, 33. Zhang *et al*. 34 discovered that the expression level of miR‐26a in EAC tissues was lower than that in non‐tumour mucosa, and miR‐26a expression was much lower in metastatic lymph nodes than in primary EAC tumours. Down‐regulation of miR‐26a leads to cell cycle arrest but it has little impact on invasion, migration and apoptosis 34. MiR‐26a suppression is favourable for the subsequent metastasis of EAC cells as well as acquisition of anoikis resistance by affecting the Rb1/E2F1 pathway 34.

Further studies are needed to explore the promising potential of miRNAs as diagnostic and prognostic biomarkers for BE/EAC, particular in the development of biomarkers that circulate in biofluids. In oesophageal SCC, several diagnostic and prognostic miRNA‐based markers derived from saliva and blood are available. Similar studies should be performed for EAC 35. For reasons of convenience and low costs for testing, compared with markers in tissues, circulating markers are more suitable for population BE/EAC screenings. Future studies are necessary to find and validate circulating markers in larger samples to develop diagnostic methods for population screening with risks of EAC development and progression 35.

## miRNAs predict EAC prognosis

Mathé *et al*. 17 discovered that low miR‐375 expression [confidence interval (CI) = 0.15–0.67, HR = 0.31; 95%] in Barrett's connected EAC tissue was highly linked with worse prognosis. Another study indicated that the pancreatic alpha‐cell mass of miR‐375‐deficient mice was decreased, and these mice were hyperglycaemic, indicating that proliferation was impaired 36. Putative miR‐375 aimed growth and proliferation of control cellular. Validated targets of miR‐375, as examined by Tarbase 37, include JAK2, Mxi1 and Ahr. MXi1 is a c‐MYC antagonist previously discovered to be overexpressed in EAC 38. A straight connection between JAK2 upon stimulation of glycine‐extended gastrin has been indicated in EA cells associated with BE 39. In Nguyen *et al*.'s 40 research, Barrett's linked EAC patients with relieved expression of miR‐375 in cancerous tissue also had bad tumour expression. Furthermore, miR‐16‐2, miR‐30e, and miR‐200a were all correlated with shorter disease‐free as well as shorter overall survival in EAC patients.

Hezova *et al*. 15 found that miR‐148 and miR‐203 were connected to overall survival and disease‐free survival in EAC patients. Survival studies indicated tremendously shorter disease‐free survival in EAC patients with up‐regulation of miR‐148 (*P* = 0.0145) and low expression of miR‐203 (*P* = 0.026) 15. Interestingly, contrasting results were obtained in ESCC patients, in which cases with down‐regulation of miR‐148 showed remarkably shorter disease‐free survival and overall survival, indicating that miR‐148 could function as an oncogene as well as tumour suppressor depending on the oesophageal cancer histological subtype 15. Analysis of regression of cox proportional hazard of global miRNA expression confirmed five unique human miRNAs that are connected with EAC patient overall survival 35. Up‐regulation of miR‐99a, miR‐143, miR‐100, miR‐199a‐3p and miR‐199a‐5p predicted poor survival 35. Increased expression of miR‐199a has also been linked with poor survival in other tumour types, including severe hepatocellular carcinoma, myeloid leucemia and cervical carcinomas 41. MiR‐99a is down‐regulated in lung cancer and considered a candidate tumour suppressor 42. In contrast, in serous ovarian carcinomas, miR‐99a is up‐regulated compared with general tissue, again showing the tissue‐specific functions of miRNAs. Skinner *et al*. 43 found differences in the levels of miR‐99b between the pathologic complete response (pCR) group and non‐pCR group. Along with miR‐99b, miR‐145, miR‐505 and miR‐451 were used to produce a score of miRNA expression profile (MEP). The numbers of low‐expressing miRNAs in MEP were largely linked with pCR. These findings proved that miR‐99 acts as an important biomarker to assess the clinical consequence in patients with EAC.

MiR‐145 has been reported as a tumour‐suppressor miRNA in various cancers, including colon, stomach, breast and lung 44. Derouet *et al*. investigated the role of miR‐145 in EAC and found a connection between shorter disease‐free survival and its expression in patients. Furthermore, the expression of miR‐145 in EAC cells had no impact on cell proliferation or response to chemotherapy drugs including 5‐FU or cisplatin but miR‐145 expression improved healing of wound cell resistance to anoikis, cell adhesion to fibronectin and cell invasion 45. In contrast, in an ESCC cell line, miR‐145 represses cell proliferation and improves anoikis, showing that it exerts specific functions depending on the oesophageal cancer histological subtype 45.

## miRNAs associated with neoadjuvant therapy

Many studies have focused on the roles of miRNAs in the chemotherapy resistance of cancer. miRNAs play critical roles in regulating apoptosis, proliferation and differentiation. The current clinical treatment options for EAC include surgery, chemotherapy and radiotherapy. The prognosis of patients receiving surgery alone is still poor, and multimodal treatment improves the survival 46. Survival rates following esophagectomy plus neoadjuvant therapies are increasing to 30–45% for 5‐year survival 47. However, the response rate to chemotherapy, including 5‐FU, is still lower than 50% 48. Chemoresistance is thus seen as a major obstacle in the effective treatment of EAC.

### MiR‐145

MiR‐145, a tumour‐suppressor miRNA, was found to be up‐regulated eightfold after induction of chemoradiotherapy 49, and the expression levels of miR‐145 were related to a shorter disease‐free survival time. Anoikis‐resistance, one of the key steps during cancer progression, helps tumour cells migrate with the bloodstream, leading to distant metastasis 50. Derouet *et al*. 45 found that miR‐145 expressing cells could form more colonies after suspension culture for 72 hrs compared with controls. To explore whether miR‐145 could protect cells against anoikis, an important form of apoptosis that enhances cell survival, they further explored anoikis levels in EAC cells and found that miR‐145 expression delayed the cleavages of poly‐ADP‐ribose polymerase (PARP) and caspase‐3, protecting EAC cells from anoikis 45. The authors further explored whether miR‐145 expression was related to cisplatin or 5‐FU resistance and found that different cell lines exhibited different resistance‐related properties 45. MiR‐145 could not protect OE33 cells from either 5‐FU or cisplatin, but could protect SK‐GT‐4 cells from cisplatin but not 5‐FU. In FLO‐1‐cells, miR‐145 could enhance cisplatin effects but had no effect on 5‐FU 45. In addition to anoikis resistance, miR‐145 could also enhance cell invasion, cell adhesion to fibronectin and wound healing.

### MiR‐221

Using four pairs of EAC‐cell lines and corresponding 5‐FU‐resistant variants, Wang *et al*. 51 found that miR‐221 was overexpressed in 5‐FU‐resistant EAC cells and human EAC tissues. Additionally, transient knock‐down of miR‐221 expression restored sensitivity of OE33‐5Fu_res_ cells to 5‐FU leading to an increased percentage of dead OE33‐5Fu_res_ cells after re‐introduction of 2.5 and 20 mg/ml 5‐FU treatment (4.7‐fold and 1.5‐fold, respectively) 51. DKK2 was identified as one of the miR‐221 target genes. Knockout of miR‐221 in 5‐FU‐resistant EAC‐cells led to decreased cell proliferation, restored chemosensitivity and increased apoptosis, and the inactivation of the b‐catenin/Wnt pathway was regulated by alterations of DKK2 levels. In addition, with endothelial mesenchymal transition (EMT) being associated with the development of 5‐FU resistance in EAC cells, miR‐221 reduction caused alterations of EMT‐related genes like vimentin and E‐cadherin, as well as a significantly lower tumour growth rate. Together this suggests that MiR‐221 might function as a prognostic biomarker and a therapeutic target for patients with 5‐FU‐resistant EAC 51.

### MiR‐21

Mir‐21 is also expressed differentially in patients that received neoadjuvant chemoradiotherapy compared with those that have not received neoadjuvant chemoradiotherapy 17. Thus, up‐regulation of miR‐21 in ECA may either confer similar molecular traits on EAC to those documented in the literature or provide selective advantages to cells within a metaplastic columnar epithelium, increasing the likelihood of neoplastic development 52, 53. Whether miR‐21 is actively secreted from cancer cells through exosome pathways or released into circulation continuously through cell apoptosis is still unclear.

### miRNA expression profile (MEP) score

A single miRNA can regulate hundreds of targets, resulting in various effects on multiple cellular functions. Based on previous small studies linking individual miRNA expressions to outcome in EAC, Skinner *et al*. created a replicable predictive model that can predict pCR to chemoradiotherapy in EAC using miRNA expressions. In their study, four miRNAs (mir‐145, mir‐451, mir‐99b and mir‐505) were united to generate a MEP score, providing a robust platform to identify patients who are highly favourable candidates for chemoradiotherapy alone as well as those for whom surgeries might be appropriate and for whom alternate therapies are recommended 43.

## Circulating miRNAs in ECA

Several studies have focused on detecting miRNAs circulating in extracellular environments, like cerebrospinal fluid, seminal fluid, saliva, tears, urine, plasma and sera 54. Although the exact roles for these kinds of miRNAs remain unclear, researchers generally believe that at least some of these miRNAs serve as mediators or regulators of endocrine or paracrine signalling. Circulating miRNAs are emerging as one of the novel non‐invasive biomarkers for cancer diagnoses due to high stability and specificity 54. miRNAs circulating in plasma have been investigated as potential biomarkers to assist with diagnosing cancers. For example, in breast cancer, specific circulating miRNAs could be adopted to differentiate normal states from cancer states and reflect the responses to chemotherapy or radiotherapy 55. This strategy would be helpful to use circulating miRNAs for population screening and to help detect EAC in patients with BE. However, the expressions of circulating miRNAs in plasma of patients with EAC or BE have not yet been sufficiently studied. To date, only three studies have explored the circulating miRNA profile in EAC.

Zhang *et al*. 56 profiled circulating miRNAs in serum of patients with EAC using Solexa sequencing analysis and identified 96 up‐regulated and 99 down‐regulated miRNAs in EAC patients compared with healthy individuals. Quantitative reverse transcription–PCR verified that the concentrations of circulating miR‐25‐3p and miR‐151a‐3p were significantly elevated in EAC patients compared with healthy controls, while the concentrations of miR‐100‐5p and miR‐375 were significantly decreased. Thus, these four miRNAs may potentially serve as a serum marker to identify patients with EAC, and circulating miRNA profiling may be clinically useful for early detection or treatment response in EAC patients 56.

Chiam *et al*. 57 investigated the diagnostic potential of circulating miRNAs for EAC. In their study, 758 miRNAs were analysed in serum‐circulating exosomes from a cohort of 18 EAC patients, 10 BE patients and 19 normal controls. miRNA expressions were measured with all possible permutations of miRNA ratios per individual. A total of 408 miRNA ratios were differentially expressed in patients with EAC compared to BE and controls (*P *< 0.05). The 179/408 ratios discriminated EACs from normal controls and BEs (*P *< 0.05) 57. A multibiomarker panel (miR‐17‐5p/miR‐194‐5p, miR‐30a‐5p/miR‐324‐5p, let‐7e‐5p/miR‐15b‐5p, miR‐25‐3p/miR‐320a, and RNU6‐1/miR‐16‐5p) revealed enhanced sensitivity and specificity over single miRNA ratios to distinguish EACs from controls or BEs 57.

Bus *et al*. 52 performed miRNA expression profiling using plasma by qPCR arrays from six healthy individuals and eight BAC and eight BE patients. The most informative diagnostic panel for distinguishing BE from controls was the combination of miRNA‐451a, miRNA‐136‐5p, miRNA‐194‐5p and miRNA‐95‐3p, with the highest sensitivity (78.4%) and specificity (85.7%). The combination of miRNA‐451a, miRNA‐382‐ 5p and miRNA‐133a‐3p had the highest accuracy for distinguishing EAC from controls. The combination of miRNA‐382‐5p, miRNA‐136‐5p and miRNA‐133a‐3p was found to have the highest potential for distinguishing EACs from BEs, with a sensitivity of 81.0% and specificity of 78.4% 52. These studies highlight the potential for miRNAs circulating in serum as markers to detect EACs.

## Conclusions

The EAC occurrence rate has been increasing over the past three decades. Underlying the development and progression of EAC and understanding the distinctive mechanisms of molecular mechanisms symbolized an irreplaceable procedure for the advancement of detailed therapeutic measures and diagnostic of EAC. miRNAs are small non‐coding RNAs that act as master regulators in multiple pathological and physiological processes and cancers. Research has demonstrated the dysregulation of miRNAs in EAC using real‐time PCR as well as array‐based miRNA profiling. However, research on the functions of these deregulated miRNAs in EAC is still limited. Moreover, many reports have established the potential use of miRNAs as prognostic and novel diagnostic markers. Studies to determine the clinical potential of miRNA‐based therapeutics and diagnostics in EAC are still needed.

## Conflicts of interest

We have no conflicts of interests.

## Funding source

This study was supported by China Scholarship Council of the Ministry of Education, P. R. China. This study was supported by Peking Union Medical College Youth Research Funds (2016) (Project No. 3332016010; Grant recipient: Xiangyi Kong) and Peking Union Medical College Graduate Student Innovation Fund (2015) (Project No. 2015‐1002‐02‐09; Grant recipient: Xiangyi Kong). The funders had no role in study design, data collection and analysis, decision to publish or preparation of the manuscript.
